# Self-Efficacy and the Maintenance of Exercise: A Quantitative Study Among Rural Older Adults

**DOI:** 10.1093/geroni/igaf122.2750

**Published:** 2025-12-31

**Authors:** Bryant O’Leary, Tasha Shaffer, Dawn Tarabochia, Brianna Routh

**Affiliations:** Montana State University, Bozeman, Montana, United States; Montana State University, Bozeman, Montana, United States; Montana State University, Bozeman, Montana, United States; Montana State University, Bozeman, Montana, United States

## Abstract

Despite the well-documented health benefits of physical activity, research shows increased sedentary behaviors among older adults (Fanning, Nicklas, & Rejeski, 2022; Leung, Sum, & Yang, 2021). A common approach to understanding exercise behaviors in high-risk populations, such as rural older adults, is to examine self-efficacy. Self-efficacy is a strong predictor of an individual’s confidence in their ability to engage in exercise (Szczuka et al., 2019). This study aimed to evaluate whether older adults with more exercise facilitators also report higher self-efficacy scores. Data were collected through a survey designed to explore self-efficacy and facilitators related to physical activity. The survey questions were developed to incorporate a previously tested exercise self-efficacy measure along with a list of facilitators and barriers previously identified in research (Bethancourt et al., 2014; Patei, Sheth, & Jain, 2019). A descriptive quantitative approach was employed for data analysis to conduct standard frequency, ANOVA tests, and multiple linear regressions exploring the relationship between self-efficacy and facilitators. The results indicated that individual preferences were significantly associated with self-efficacy (p<.001). This research underscores the critical role of individual preferences in fostering exercise habits, particularly among rural older adults. Factors such as enjoyment of exercise, belief in its importance, feelings of guilt when inactive, awareness of its benefits, and the integration of exercise into daily routines all contribute to shaping exercise behaviors. These findings are pivotal in developing effective strategies to promote long-term exercise habits, emphasizing the need to consider personal preferences when designing interventions for older adults in rural communities.

